# *Poria cocos* polysaccharide enhances the persistence and anti-colitic efficacy of three-strain probiotic preparation in DSS-induced colitis in mice

**DOI:** 10.3389/fmicb.2026.1942804

**Published:** 2026-10-29

**Authors:** Tingyu Li, Yan Wang, Chong Wang

**Affiliations:** 1 Dalian Women and Children's Medical Group, Dalian, China; 2 Institute of Integrative Medicine, Dalian Medical University, Dalian, China; 3 Department of Clinical Pharmacology, College of Pharmacy, Dalian Medical University, Dalian, China

**Keywords:** gastrointestinal persistence, inflammatory bowel disease, live Combined Bifidobacterium and Lactobacillus tablets, plant polysaccharides, *Poria cocos* polysaccharide

## Abstract

**Introduction:**

Probiotic supplementation is limited within the gut. This study aimed to screen potential prebiotic candidates that promotes the gastrointestinal persistence of probiotics from plant polysaccharides, and evaluate its potentiation on probiotics in inflammatory bowel disease (IBD) treatment.

**Methods:**

The effects of natural polysaccharides on the intestinal survival of Golden Bifid (GB, a three-strain probiotic preparation including *Bifidobacterium longum, Lactobacillus bulgaricus*, and *Streptococcus thermophilus*) were investigated through in vitro proliferation assay, stress resistance analysis, and fecal abundance determination in mice. The anti-colitic effect of GB and prebiotic candidate in IBD were evaluated in dextran sulfate sodium (DSS) -induced colitis in wild-type mice and antibiotic-treated (ABX) mice.

**Results:**

*Poria cocos* polysaccharide (PcP) significantly promoted the proliferation of *B. longum, L. bulgaricus*, and *S. thermophilus*, and improved their tolerance to gastric and intestinal juice, and bile salts. After co-administration with PcP, their abundance in feces was increased, and their decline in fecal abundance after withdrawal was delayed. Importantly, the combined use of PcP significantly enhanced the anti-colitic effect of GB on colitis. Furthermore, in ABX mice, PcP alone failed to alleviate colitis, but still enhanced the anti-colitic effect of GB.

**Discussion:**

PcP promotes the intestinal persistence of probiotics, thereby enhancing their intestinal protective effects. This study provides a novel application for improving the anti-colitic efficacy of probiotics by combination with PcP.

## 1 Introduction

In recent years, the critical role of the gut microbiota in maintaining human metabolic homeostasis and balance has been increasingly recognized through deep sequencing studies of large-scale disease control cohorts (Yan et al., 2024; Lee et al., 2022). The gut microbiota, composed of a complex community of microorganisms, participates in various physiological processes such as nutrient absorption, immune regulation, and metabolic conversion, and its dysbiosis is closely associated with the occurrence and development of numerous diseases, especially inflammatory bowel disease (IBD) (Yang and Cong, 2021; Rooks and Garrett, 2016; Hou et al., 2022; Ni et al., 2017; Lavelle and Sokol, 2020). IBD, including Crohn's disease and ulcerative colitis, is a chronic recurrent inflammatory disorder of the gastrointestinal tract with unclear pathogenesis, and it has become a global public health challenge due to its increasing incidence and difficulty in treatment (Kaplan, 2015). In clinical practice, fecal microbiota transplantation (FMT) and supplementation of specific probiotics have emerged as effective therapeutic strategies for IBD, as they can reconstruct the disturbed gut microbiota homeostasis and thereby alleviate intestinal inflammation (Yan et al., 2024; Wu et al., 2025; Tian et al., 2024; Huo et al., 2022).

Golden Bifid (GB), a commercial three-strain probiotic preparation, is widely used in China for the clinical treatment of various gastrointestinal disorders (Zhu et al., 2017). This probiotic preparation is composed of three core beneficial strains, namely *Bifidobacterium longum, Lactobacillus bulgaricus*, and *Streptococcus thermophilus*, which have been proven to exert significant protective effects against infantile diarrhea, irritable bowel syndrome, slow transit constipation, and ulcerative colitis (Liu et al., 2025; Luo et al., 2022; Yao et al., 2017). GB can regulate the balance of gut microbiota, inhibit the growth of pathogenic bacteria, enhance the integrity of the intestinal barrier, and reduce intestinal inflammation (Liu et al., 2025; Luo et al., 2022; Yao et al., 2017), making it a promising probiotic candidate for the treatment of IBD. However, the clinical application effect of GB is still limited by the low colonization efficiency of exogenous probiotics in the intestinal tract.

A major challenge faced by exogenous probiotic supplementation is the harsh gastrointestinal environment, including low pH in gastric juice, high concentration of bile salts in the intestinal tract, and the competition from inherit gut microbiota, which significantly reduce the survival rate and colonization ability of probiotics, thereby weakening their biological activity and protective efficacy (Huang et al., 2022; Lynch et al., 2022; Pedrolli et al., 2019). Therefore, improving the intestinal colonization efficiency of exogenous probiotics has become a key focus in the field of probiotic research. Plant polysaccharides, as natural prebiotics derived from various plants, have attracted extensive attention due to their safety, non-toxicity, and excellent prebiotic activity (Wu et al., 2025; Jin et al., 2026). They can be selectively utilized by probiotics as carbon sources to promote their proliferation, enhance their tolerance to adverse environments, and ultimately improve their intestinal colonization efficiency (Wu et al., 2025; Xing et al., 2017; Yu et al., 2025).

Based on the above background, this study employed the three-strain probiotic preparation as the model probiotic and established probiotic culture model *in vitro* and mouse model of intestinal persistence *in vivo*. A rapid screening and systematic characterization of multiple plant polysaccharides were carried out to identify potential prebiotics that can promote the intestinal persistence of *B. longum, L. bulgaricus*, and *S. thermophilus*. Furthermore, a mouse model of DSS-induced colitis was utilized to directly investigate the enhancing effect of plant polysaccharides on the biological activity of GB in alleviating colitis. Our findings revealed that *Poria cocos* polysaccharide (PcP) can significantly promote the intestinal persistence of GB and enhance its protective effect against colitis, providing preliminary evidence for its potential application value as a potential prebiotic candidate. This research not only provides a theoretical basis and experimental support for the development of a new generation of “prebiotic + probiotic” products but also offers a novel strategy for improving the protective efficacy of probiotics in IBD treatment.

## 2 Materials and methods

### 2.1 Materials

Golden Bifid (GB, Live Combined Bifidobacterium and Lactobacillus tablets) was purchased from Inner Mongolia Shuangqi Pharmaceutical Co., Ltd., which contains three core probiotic strains *Bifidobacterium longum, Lactobacillus bulgaricus*, and *Streptococcus thermophilus*. A total of 100 commercially available plant polysaccharides, including *Poria cocos* polysaccharide (PcP, Batch number: S35809), were purchased from Yuan Ye Biotechnology Co., Ltd. (Shanghai, China). PcP is a grayish-white powder with a drying weight loss of less than 5% and has a polysaccharide content of ≥50% or ≥80%. Dextran sulfate sodium (DSS, molecular weight 36,000–50,000 Da) was obtained from Meilun Biotechnology Co., Ltd. (Dalian, China). All other reagents and chemicals used in this study were of analytical grade and commercially available unless otherwise specified.

### 2.2 Animals and ethical approval

Specific pathogen-free (SPF) C57BL/6 mice (6–8 weeks old, male, weighing 20–22 g) were purchased from Liaoning Changsheng Biotechnology Co., Ltd. All mice were housed in a standardized animal facility under controlled conditions (temperature: 22 ± 2 °C, relative humidity: 50 ± 5%, 12 h light/dark cycle) with free access to sterile water and standard chow diet. All animal experimental protocols were reviewed and approved by the Animal Ethics Committee of Dalian Medical University (Approval No.: AEE24313), and all procedures were performed in accordance with the Guidelines for the Care and Use of Laboratory Animals.

Mice were numbered based on their baseline body weight and randomly assigned to experimental groups using a computer-generated random number generator before the initiation of treatment. Investigators were blinded to group allocation when performing DAI scoring and histological evaluation of colon tissues; sample identifiers were anonymized to conceal group information during these assessments. Blinding was not performed during sample processing and subsequent analytical measurements (including qPCR and SCFA quantification), as experimental workflow and batch preparation required knowledge of sample grouping.

### 2.3 *In vitro* probiotic culture and growth assays

All three probiotic strains isolated from GB were cultured under anaerobic conditions at 37 °C in MRS broth (for *Lactobacillus bulgaricus* and *Streptococcus thermophilus*) or Bifidobacterium broth (for *Bifidobacterium longum*). For the growth curve assay, probiotics were inoculated into broth supplemented with different plant polysaccharides (1 mg/ml, unless otherwise specified) at an initial inoculum concentration of 1% (v/v). The absorbance at 600 nm (OD) was measured at the designated time points using a microplate reader (BioTek, USA) to draw the growth curve. Each experiment was performed in triplicate.

### 2.4 Caco-2 adhesion assay

Adhesion of bacterial strains to Caco-2 cells was determined as follows. Briefly, Caco-2 cells were seeded in 12-well plates at a density of 1 × 10^5^ cells/mL and cultured in DMEM supplemented with 10% fetal bovine serum and 1% antibiotic-antimycotic solution at 37 °C under 5% CO_2_ for 14 days to allow differentiation. Bacterial strains were cultured for 18 h, harvested by centrifugation (4000 × g, 20 min), washed twice with PBS, and resuspended in antibiotic- and serum-free DMEM to 1 × 10^8^ CFU/mL. The bacterial suspension was added onto Caco-2 monolayers and incubated for 2 h at 37 °C with 5% CO_2_. Non-adherent bacteria were removed by three washes with PBS. Cells were lysed using PBS containing 0.1% (v/v) Triton X-100. The number of adherent bacteria was quantified by plate-counting. Adhesion ability was calculated as the percentage of adherent bacteria relative to the initially added bacterial count.

### 2.5 Short-chain fatty acids (SCFAs) and lactic acid (LA) determination

Bacterial suspensions were plated and incubated for 24 h with or without PcP. Cell-free supernatants were collected by centrifugation (10 000 × g, 4 °C, 20 min). Sample supernatants were derivatized using aniline-EDAC system at 4 °C for 2h, quenched, and subjected to LC-MS/MS analysis. Chromatography was performed using a Phenomenex C18 column (3.0 × 100 mm, 3.0 μm, 40 °C). Mobile phase A: 0.1 % formic acid in water; mobile phase B: acetonitrile. Gradient: 0–1.0 min 20 % B; 1.0–2.5 min 40 % B; 2.5–3.0 min 90 % B; 3.0–4.0 min 20 % B; 4.0–5.5 min 20 % B, flow rate 0.3 mL/min. A triple-quadrupole mass spectrometer was operated under MRM positive-ion mode. The monitored precursor-product ion pairs for aniline-derivatized metabolites were as follows: acetic acid (m/z 136.1 → 94.0 / 77.0), propionic acid (m/z 150.1 → 94.0 / 77.0), butyric acid (m/z 164.1 → 94.0 / 77.0), and lactic acid (m/z 152.1 → 108.0 / 77.0). Absolute concentrations were calculated against external calibration curves.

### 2.6 *In vivo* probiotic persistence assay

Mice were randomly divided into two groups: control (GB alone) group and GB + PcP combination group. Mice in the control group were orally administered GB (10^8^ CFU/mouse) once daily for 1 week. Mice in the combination group were orally administered GB (10^8^ CFU/mouse) and PcP (200 mg/kg body weight) once daily for 1 weeks. PcP administration was continued for an additional week after GB withdrawal. Fresh fecal samples were collected from each mouse before GB administration and at 1, 2, and 3 weeks after the start of GB administration.

Total bacterial DNA was extracted from fecal samples using a Fecal DNA Extraction Kit according to the manufacturer's instructions. The concentration and purity of the extracted DNA were detected using a Nanodrop spectrophotometer (Thermo Fisher Scientific, USA). The abundance of the three probiotic strains in fecal samples was quantitatively analyzed by quantitative real-time polymerase chain reaction (qPCR) as described below.

### 2.7 Establishment of DSS-induced colitis model and drug administration

A mouse model of colitis was induced by administering 3% (w/v) DSS in drinking water ad libitum for 7 consecutive days. Mice in the normal control group were given sterile drinking water without DSS. For the anti-colitic intervention, mice were randomly divided into five groups: normal control group, DSS model group, PcP alone group, GB alone group, and GB + PcP combination group. Each group contained six mice.

Mice were orally administered PcP (200 mg/kg body weight), or GB (10^8^ CFU/mouse), or PcP/GB once daily for 14 consecutive days. All drug administrations were started 1 week before DSS induction.

During the experiment, the water consumption (Supplementary Table 1), body weight, diarrhea status, and rectal bleeding of mice were recorded daily to calculate the Disease Activity Index (DAI) according to the standard scoring system (body weight loss: 0–4 points; diarrhea: 0–4 points; hematochezia: 0–4 points, total DAI score range: 0–12 points). After the last administration, mice were fasted overnight. Subsequently, FITC-dextran (Sigma, average mol wt 3,000–5,000) was administered by gavage at 200 mg/kg (0.2 ml per mouse), and blood samples were collected 4 h later to detect the fluorescence intensity of FITC-dextran. For the FITC-dextran determination, serially diluted FITC-dextran standards were used to build a standard curve. Fluorescence was read on a microplate reader (Ex = 485 nm, Em = 520 nm). Serum FITC-dextran concentrations were calculated against this standard curve.

Then, mice were sacrificed and the colon was then dissected, its length was measured, and the appearance was photographed. Colonic tissue samples were collected, fixed in 4% paraformaldehyde for hematoxylin-eosin (HE) staining, or stored at −80 °C for subsequent inflammatory factor detection. Histopathological scoring was performed by experienced researchers who were blinded to experimental group assignments. Three parameters were assessed: inflammation (0–3), crypt damage (0–5), and ulceration (0–3). The total histological score was calculated as the sum of these three individual sub-scores. Detailed scoring criteria are summarized in Supplementary Table 2.

For antibiotic-treated (ABX) mice, a quadruple antibiotic cocktail regimen was used to deplete the gut microbiota. Briefly, mice were given drinking water containing the antibiotic cocktail (ampicillin, 1 g/L; neomycin, 1 g/L; metronidazole, 1 g/L; vancomycin, 0.5 g/L) ad libitum for 7 consecutive days before DSS induction. After gut microbiota depletion, the subsequent colitis induction and drug administration protocols were the same as described above.

### 2.8 Quantitative qPCR analysis

Total RNA was extracted from colonic tissue samples using Trizol reagent (Invitrogen, USA) according to the manufacturer's instructions. Complementary DNA (cDNA) was synthesized from 1 μg of total RNA using a Reverse Transcription Kit (Takara, Japan). qPCR was performed using a SYBR Green qPCR Master Mix (Takara, Japan) on a StepOnePlus Real-Time PCR System (Applied Biosystems, USA) to detect the mRNA expression levels of inflammatory factors (IL-1β, TNF-α, IL-6, and INF-γ). GAPDH was used as an internal reference gene for colonic tissue samples.

For the detection of probiotic abundance in fecal samples, qPCR was performed using specific primers for *B. longum, L. bulgaricus*, and *S. thermophilus*. The qPCR results are presented as relative abundance normalized to total bacterial 16S rRNA., which were further normalized to the control group (set as 1) to show fold changes of target strains.

The relative expression levels were calculated using the 2^−Δ*ΔCT*^ method. All primers used in this study were synthesized by Sangon Biotechnology Co., Ltd. (Shanghai, China), and the primer sequences are listed in Supplementary Table 3.

### 2.9 Statistical analysis

All experimental data were expressed as the mean ± standard deviation (SD). Statistical analysis was performed using GraphPad Prism 10.0 software (GraphPad Software, USA). For non-longitudinal endpoint parameters (e.g., colon length, histopathology scores measured only at the terminal sacrifice), differences between multiple groups were compared using one-way analysis of variance (ANOVA) followed by Tukey's *post-hoc* test. Longitudinal data including body weight, DAI, diarrhea, bleeding scores and fecal probiotic abundance were analyzed by linear mixed-effects models, with mouse ID set as random intercept, treatment and time as fixed effects. *P* values < 0.05 were considered statistically significant.

## 3 Results

### 3.1 *Poria cocos* polysaccharide (PcP) promotes probiotic growth as a natural prebiotic candidate

To identify prebiotic candidates from plant polysaccharides, 100 commercially available crude plant polysaccharides were collected and used for *in vitro* culture of probiotics. The proliferation of probiotics was evaluated by monitoring the absorbance of bacterial culture medium (Figure 1A). After adding different plant polysaccharides at a concentration of 1 mg/ml, the growth of probiotics was altered to varying degrees (Figure 1B). Among these polysaccharides, those that increased probiotic growth by more than 80% were as follows: *Astragalus* polysaccharide, *Panax notoginseng* polysaccharide, PcP, and *Dipsacus asper* polysaccharide for *B. longum*; tea polysaccharide, PcP, *Fomes officinalis* polysaccharide, and longan polysaccharide for *L. bulgaricus*; and PcP, aloe polysaccharide, cinnamon polysaccharide, and *Achyranthes bidentata* polysaccharide for *S. thermophilus*. Notably, PcP exhibited a strong proliferative-promoting effect on all three probiotic strains (Figure 1C). Therefore, PcP was selected as a potential prebiotic component for subsequent studies.

**Figure 1:**
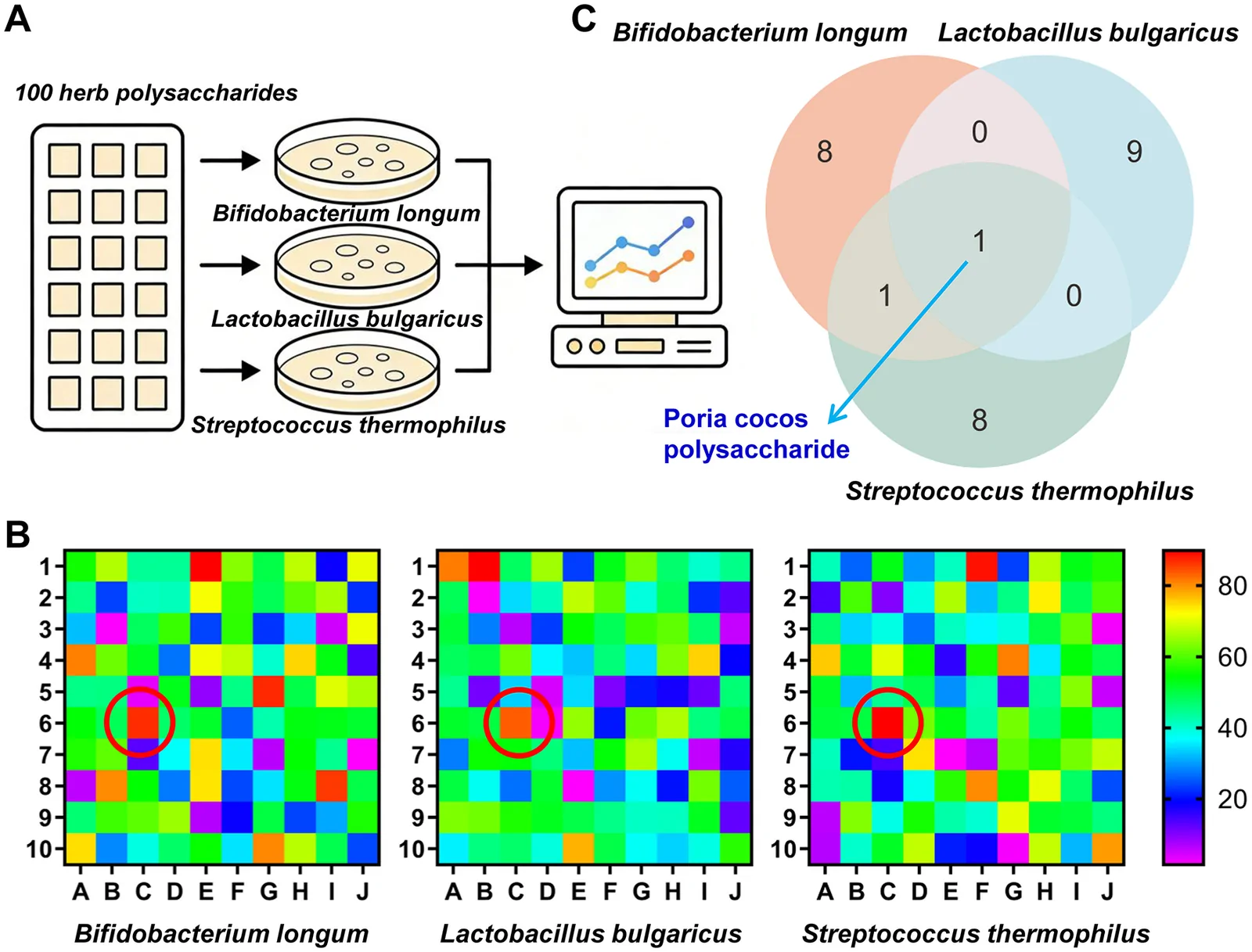
Screening of natural polysaccharides that promote probiotic proliferation. **(A)** Schematic diagram of the experimental workflow. **(B)** Heatmap showing the effects of 100 natural polysaccharides on the *in vitro* proliferation of *Bifidobacterium longum, Lactobacillus bulgaricus*, and *Streptococcus thermophilus*. The red circle indicates *Poria cocos* polysaccharide (PcP). *N* = 3. **(C)** Venn diagram of the top 10 natural polysaccharides that promote probiotic proliferation.

To further evaluate the application potential of PcP as a probiotic synergist, we systematically investigated its effects on the growth characteristics and stress resistance of probiotics. PcP preparations with two different polysaccharide purities (≥50 % and 80%) were used for activity validation to precisely evaluate the contribution of polysaccharide fractions within this plant extract. First, the effect of PcP at a concentration of 1 mg/ml on the growth curves of probiotics was observed. The results showed that supplementation of PcP in the culture medium accelerated the growth rate of all three probiotic strains, and PcP with 80% polysaccharide purity exhibited greater activity in promoting bacterial proliferation (Figure 2A). Meanwhile, we also found that PcP (1 mg/ml, 50%) significantly increased the survival rates of the three probiotics in artificial gastric juice (pH = 3), artificial intestinal fluid (pH=8), and bile salt solution (1%) (Figure 2B). Likewise, PcP (80%) afforded superior protection to the tested strains against challenges from digestive fluids and bile salts (Figure 2B). Furthermore, Caco-2 cell adhesion assays and SCFA-production analysis were performed to evaluate the effects of PcP on the intestinal-mucosa-adhesion potential and bioactive-metabolite secretion of the probiotics. *B. longum, L. bulgaricus, and S. thermophilus* exhibited differential adhesion capacities to Caco-2 epithelial cells, reaching 3.2 %, 29.4 %, and 4.1 %, respectively. Upon co-incubation with PcP, their adhesion rates were significantly increased to 5.2 %, 48.0 %, and 6.7 %, indicating the potential for improved *in-vivo* gut persistence (Figure 2C). Moreover, three strains produced substantial amounts of metabolites dominated by acetate (AA, the major SCFA for these strains) and lactate (LA). Supplementation with PcP significantly elevated the yields of these metabolites, with relative increases of 66.3 %−74.7 % for AA, 30.0 %−56.3 % for propionate (PA), 28.0 %−49.0 % for butyrate (BA), and 11.1 %−35.0 % for LA (Figures 2D–F). These observations demonstrate that PcP enhances the metabolic activity of the tested probiotic strains *in vitro*. Collectively, these results indicate that crude PcP can promote the growth and enhance the stress resistance of probiotics.

**Figure 2:**
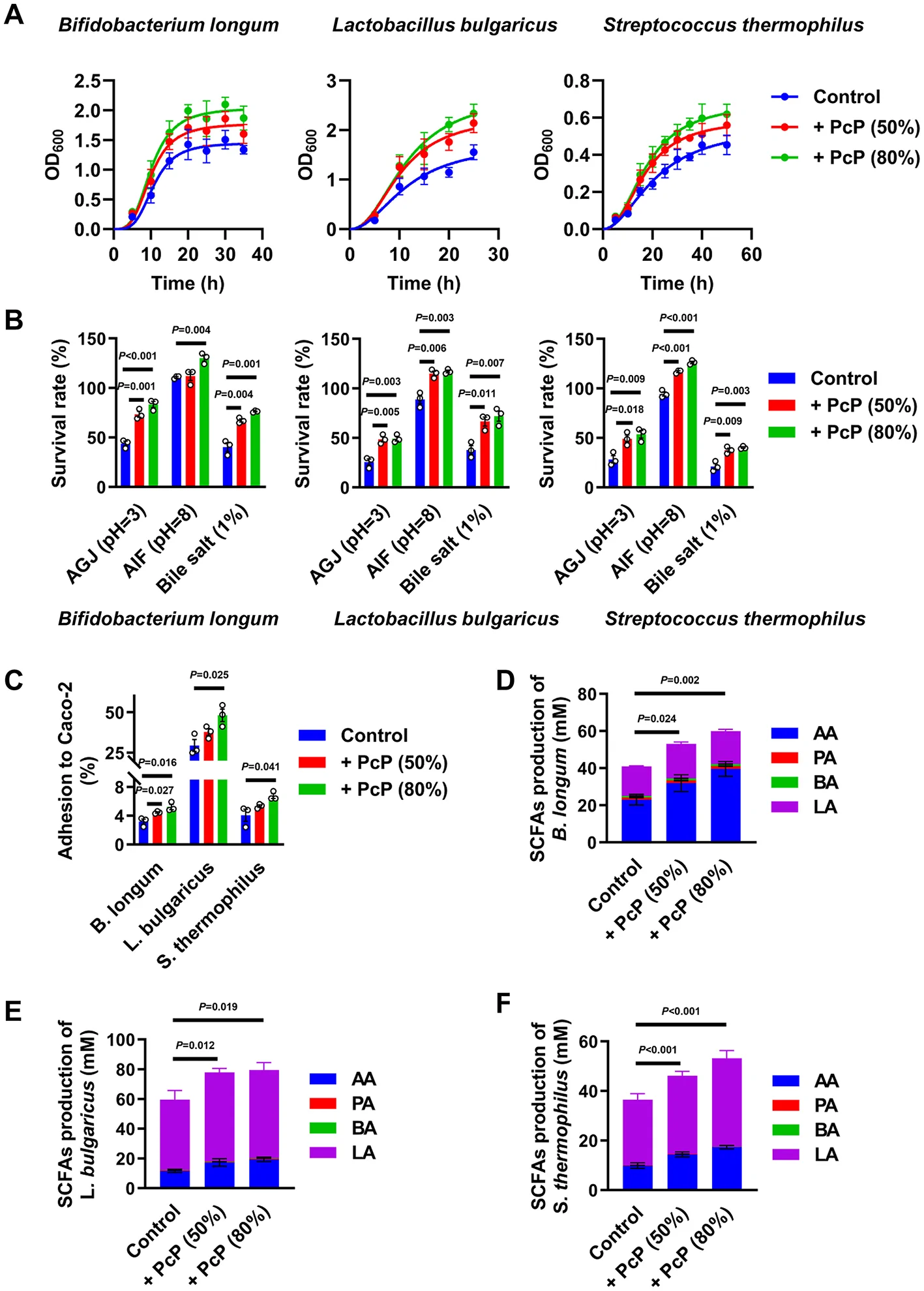
*Poria cocos* polysaccharide promotes probiotic proliferation and stress resistance. **(A)** Effect of PcP on the growth curve of probiotics. **(B)** Effect of PcP on the survival rate of probiotics in artificial gastric juice (AGJ), artificial intestinal fluid (AIF), and bile salt solution. **(C)** Effect of PcP on the Caco-2 cell adhesion rate of probiotics. **(D–F)** Effect of PcP on short-chain fatty acid (SCFA) and lactate production by probiotics. AA, acetic acid; PA, propionic acid; BA, butyric acid; LA, Lactic acid. *N* = 3. PcP, *Poria cocos* polysaccharide.

We further investigated the effect of PcP on the persistence and decline in fecal abundance after withdrawal of probiotics in the mouse intestine. Mice were orally administered three-strain probiotic preparation alone or in combination with PcP, and the abundance change curves of the three probiotics in feces were quantitatively detected at different stages (Figure 3A). The results showed that 1 week after oral administration of three-strain probiotic preparation, the abundances of the three probiotics in mouse feces were significantly increased, reaching 6.2-fold, 6.5-fold, and 6.1-fold the baseline level, respectively; after discontinuing gavage, the abundances of probiotics gradually decreased, and only 1.5-1.8-fold the baseline level two weeks after probiotic withdrawal (Figure 3B), indicating that exogenous probiotic supplementation faces the challenge of poor long-term persistence. In contrast, after combined use with PcP, the degree of increase in probiotic abundance in feces was significantly enhanced. One week after three-strain probiotic preparation supplementation, the abundances of the three probiotics were 13.2-fold, 32.4-fold, and 72.3-fold the baseline level, respectively, and 3.5-13.9-fold the baseline level remain 2 weeks after probiotic withdrawal. Throughout the observation period, the combined use of PcP significantly increased the probiotic abundance, which was 3.5–17.6-fold that of the probiotic alone group at the same period (Figure 3B). These results suggest that PcP enhances the persistence ability of probiotics in the mouse intestine, prolongs their retention time in the intestine, and may thereby enhance the protective effect of probiotics.

**Figure 3:**
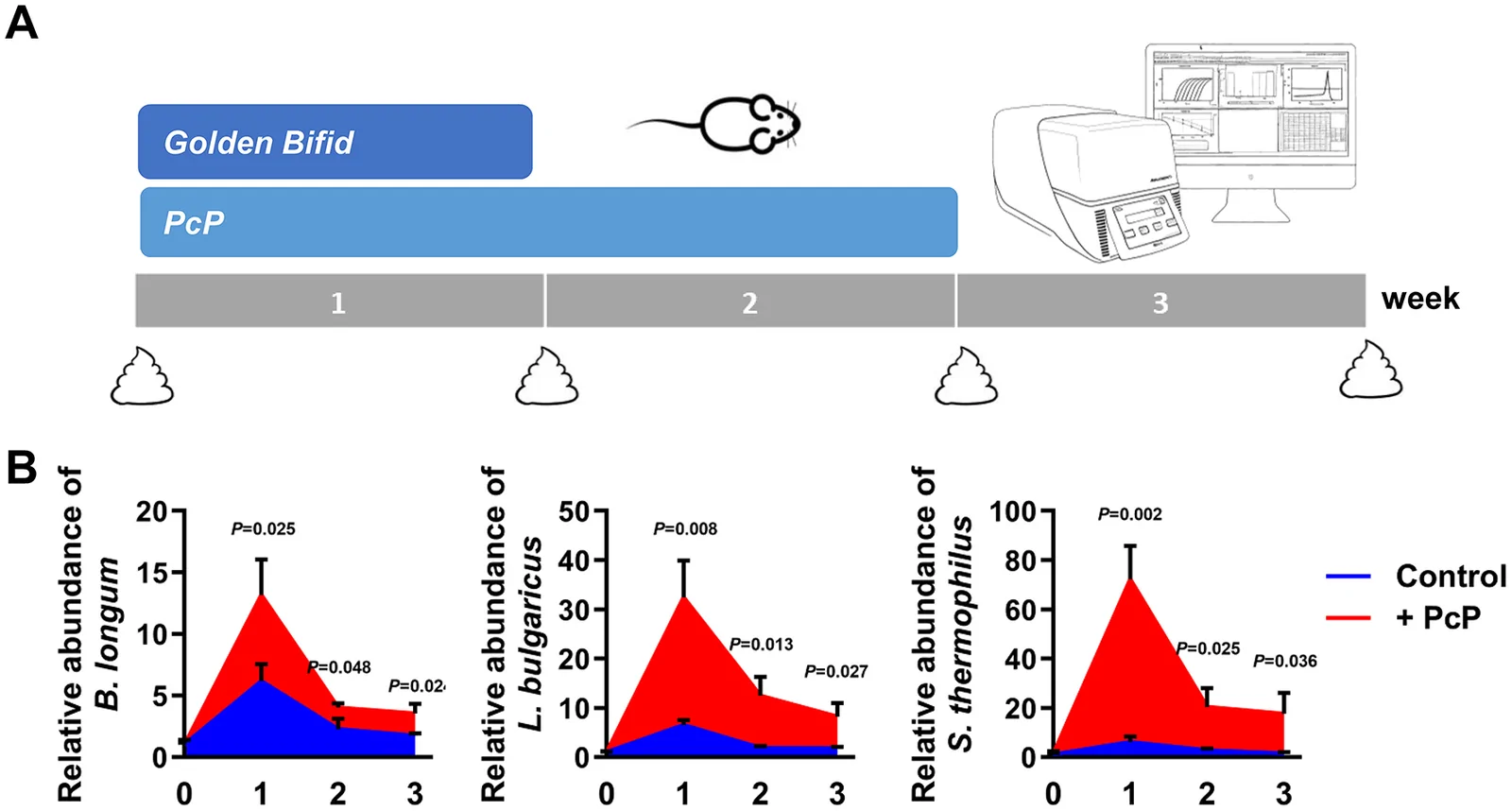
*Poria cocos* polysaccharide promotes probiotic persistence in the mouse intestine. **(A)** Animal experimental design. Mice were orally administered Golden Bifid for 1 week, or concurrently administered PcP for 2 weeks. Fecal samples were collected at 0, 1, 2, and 3 weeks after oral administration. The abundances of *B. longum, L. bulgaricus*, and *S. thermophilus* were determined by qPCR. **(B)** Relative abundances of probiotics in mouse feces. N = 3. *P* values were obtained by compared with the control group. PcP, *Poria cocos* polysaccharide.

### 3.2 PcP and three-strain probiotic preparation Alleviate DSS-Induced Colitis in Mice

To directly evaluate the effect of PcP on the anti-colitic effect of probiotics, a mouse model of DSS-induced colitis was established to investigate the effects of PcP and GB alone or in combination on colitis (Figure 4A). The results showed that both PcP and GB alone alleviated the symptoms of colitis in mice. Compared with the DSS group, gavage with PcP or GB significantly relieved DSS-induced symptoms such as diarrhea, colon bleeding, and weight loss in mice, which produced lower DAI (Figures 4B–E). Colonoscopy revealed that DSS drinking caused colonic ulcer and bleeding, loose stools, villous atrophy and fibrotic changes, while gavage with PcP or GB significantly improved colonic pathological damage (Figure 4F). Meanwhile, gavage with PcP or GB significantly reduced the plasma concentration of FITC-dextran (Figure 4G), indicating that PcP and GB improved DSS-induced intestinal barrier disruption in mice. Furthermore, DSS drinking significantly shortened the colon length; after gavage with PcP or GB, the bloody contents in the cecum and colon were reduced, and the colon length was restored (Figure 4H). Collectively, these results indicate that both PcP and GB alone exhibit activity in alleviating DSS-induced colitis in mice.

**Figure 4:**
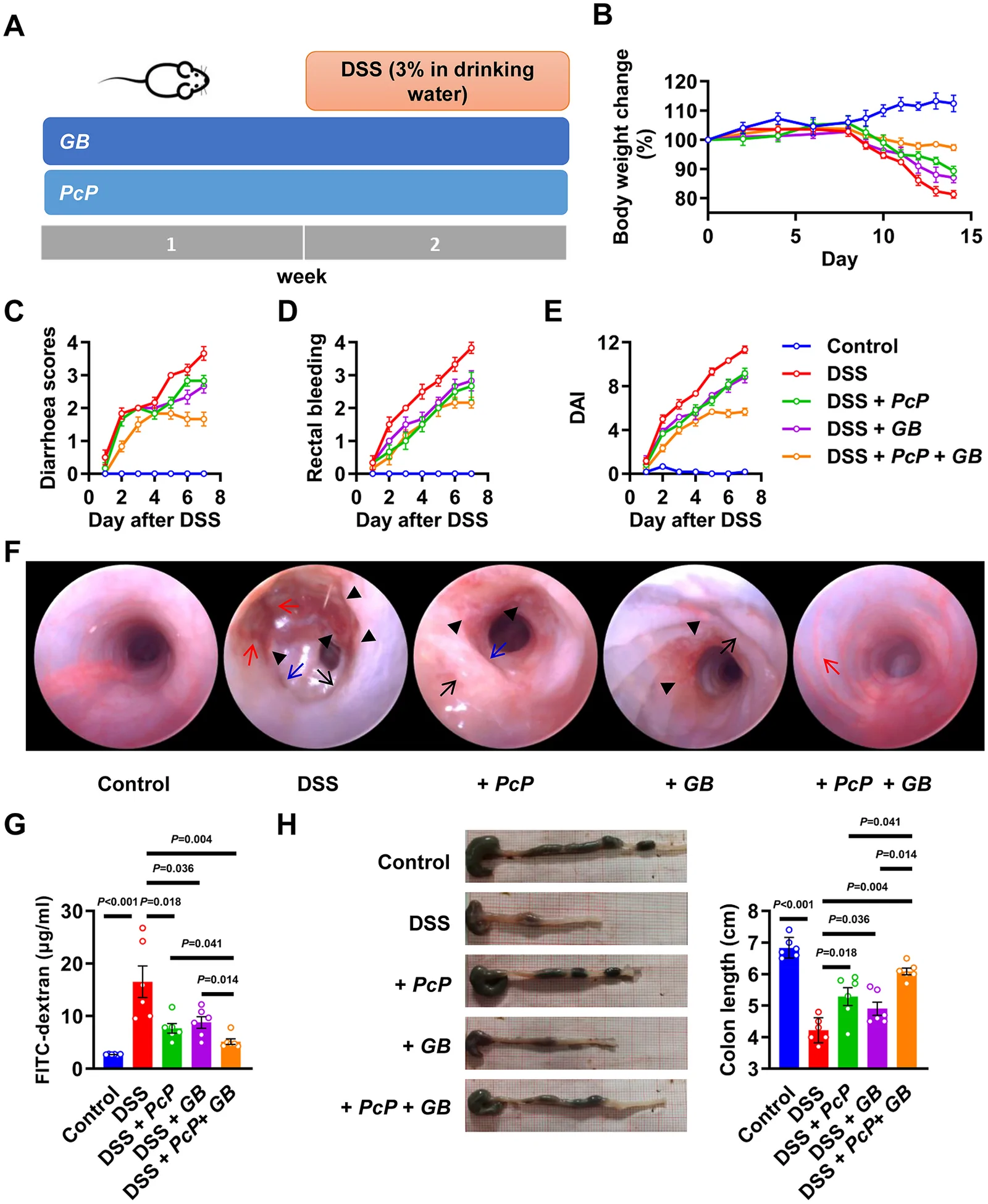
*Poria cocos* polysaccharide enhances the anti-colitic effect of probiotics on colitis in mice. **(A)** Animal experimental design. Mice were given 3% dextran sulfate sodium (DSS) in drinking water for 1 week to induce colitis, and were orally administered Golden Bifid alone or in combination with PcP 1 week in advance. **(B)** Body weight change curve. **(C)** and **(D)**. Diarrhea and rectal bleeding scores. **(E)** Disease activity index (DAI). **(F)** Colonoscopic observation. Black triangles indicate hemorrhage; blue arrows indicate ulceration; black arrows indicate villous atrophy and fibrotic changes; red arrows indicate vasodilation. DAI and colonoscopy assess different aspects of colitis; near-normal endoscopic mucosa does not exclude mild residual inflammation detected by DAI. **(G)** Plasma FITC-dextran concentration. **(H)** Colon appearance and length. *N* = 6. PcP, *Poria cocos* polysaccharide; GB, Golden Bifid.

More importantly, we observed that the combination of PcP and GB exerted an additive alleviative effect on colitis. Compared with the PcP alone or GB alone treatment groups, the combined treatment group exhibited significantly milder colitis symptoms, which were characterized by reduced diarrhea, bleeding, and body weight loss, a lower DAI, fewer colonic ulcers, less hemorrhage and loose stools, further recovery of colonic villi, decreased plasma FITC-dextran levels, and further restoration of colon length (Figures 4B–H). These results demonstrate that both PcP and GB can alleviate colitis, reduce colonic damage, and promote the recovery of intestinal barrier in mice; furthermore, their combined application significantly enhances the anti-colitic efficacy.

To further characterize the anti-colitic effects of PcP and GB, we evaluated the colonic tissue pathological damage and inflammatory levels in mice after treatment with PcP and GB alone or in combination. HE staining showed that DSS administration via drinking water caused severe colonic pathological changes, characterized by the disappearance of villous structure and massive inflammatory cell infiltration. After treatment with PcP or GB alone, the villous structure was partially restored, but inflammatory cell infiltration still existed. In the combined treatment group, the colonic villous structure was intact with only a small amount of inflammatory cell infiltration (Figure 5A and Supplementary Figure 1A). Similarly, the levels of colonic inflammatory factors (IL-1β, TNF-α, IL-6, and INF-γ) were significantly decreased after treatment with PcP or GB alone, and were further reduced after combined treatment (Figure 5B). These results further confirm that both PcP and GB can alleviate colonic pathological damage and reduce inflammatory levels in mice; moreover, their combined application enhances the anti-colitic effect.

**Figure 5:**
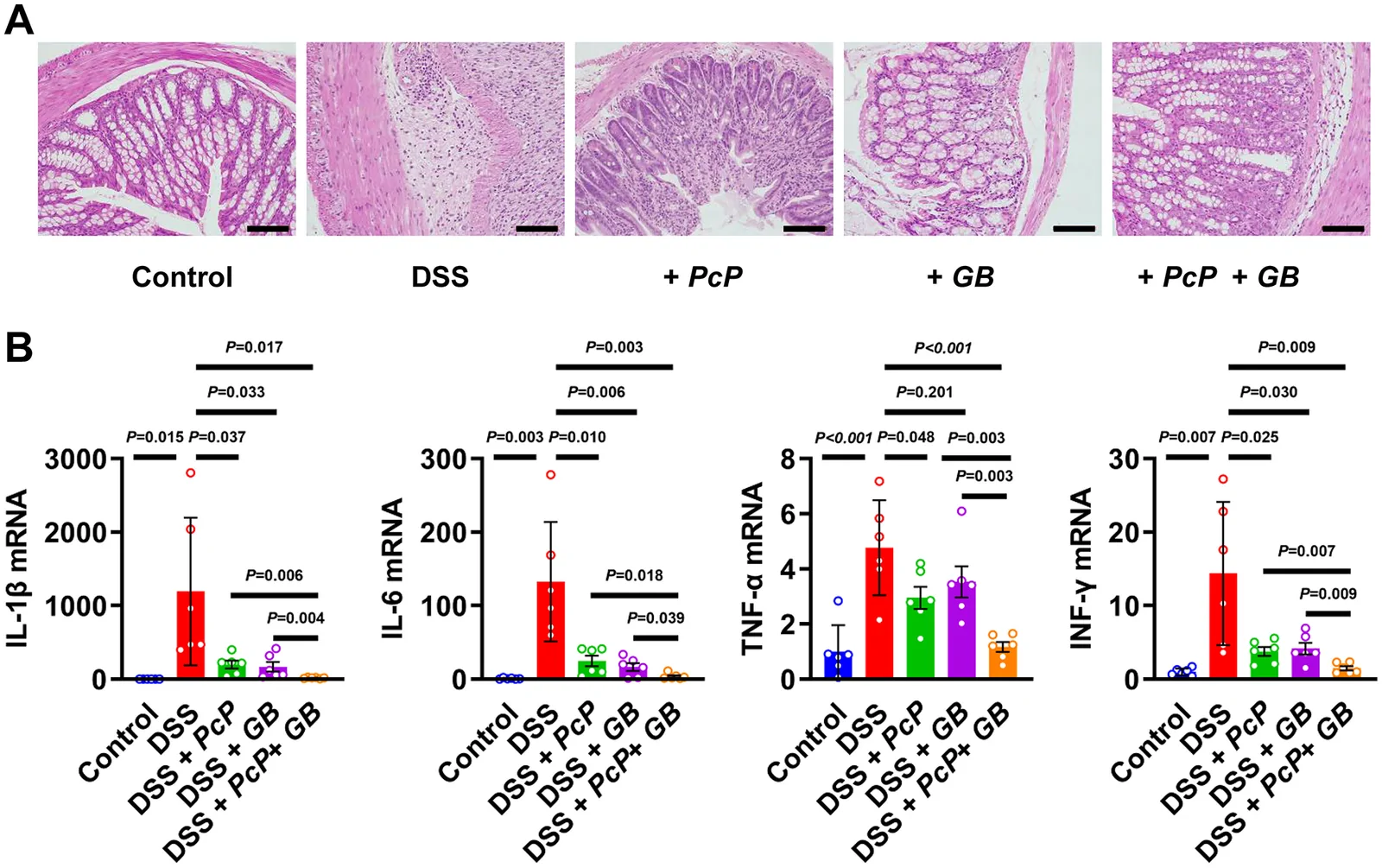
*Poria cocos* polysaccharide enhances the effect of probiotics in alleviating colonic pathological damage and inflammatory levels. **(A)** Hematoxylin-eosin (HE) staining. Scale bars: 100 μm. Magnification: 400×. **(B)** mRNA levels of IL-1β, IL-6, TNF-α, and INF-γ in colon tissues. *N* = 6. PcP, *Poria cocos* polysaccharide; GB, Golden Bifid.

### 3.3 PcP enhances the anti-colitic effect of probiotics on colitis by promoting their intestinal persistence

To rule out the influence of the inherent protective effect of PcP, we further used ABX mice with their original gut microbiota depleted by antibiotic cocktail, to investigate the anti-colitic effects of PcP and GB alone or in combination on colitis (Figure 6A). First, the fecal abundance of the three probiotic strains was quantified via qPCR to evaluate whether PcP facilitates GB persistence under colitic conditions. Compared with the DSS group, PcP treatment exerted almost no effect on the fecal abundance of the three probiotic strains. Supplementation with GB increased the abundances of the three probiotics by 77.2-fold, 124-fold, and 100-fold, respectively (Figure 6B). In the PcP + GB combination group, fecal abundances of the three probiotics were further elevated to 174-fold, 202-fold, and 248-fold relative to the DSS group (Figure 6B). This pattern recapitulates our observations in healthy mice (Figure 3B), further indicating that PcP promotes intestinal persistence of GB even under inflammatory colitis conditions. The results showed that GB alone still alleviated DSS-induced colitis symptoms; compared with the DSS group, mice in the GB treatment group exhibited significantly relieved symptoms such as diarrhea, bleeding, and body weight loss (Figure 6C). Under colonoscopy, GB treatment reduced colonic mucosal hemorrhage and loose stools (Figure 6D); the plasma FITC-dextran concentration decreased, indicating the recovery of intestinal barrier function and reduced permeability (Figure 6E); after GB supplement, the colonic bloody contents decreased and the colon length was restored (Figure 6F); the pathological tissue damage in the GB group, such as colonic villus destruction and inflammatory cell infiltration, was significantly alleviated (Figure 7A and Supplementary Figure 1B); the expression levels of inflammation-related factors (IL-1β, TNF-α, IL-6, and INF-γ) in colon tissues were significantly downregulated (Figure 7B). These results indicate that GB still exerts anti-colitis effect in antibiotic-treated mice with depleted gut microbiota.

**Figure 6:**
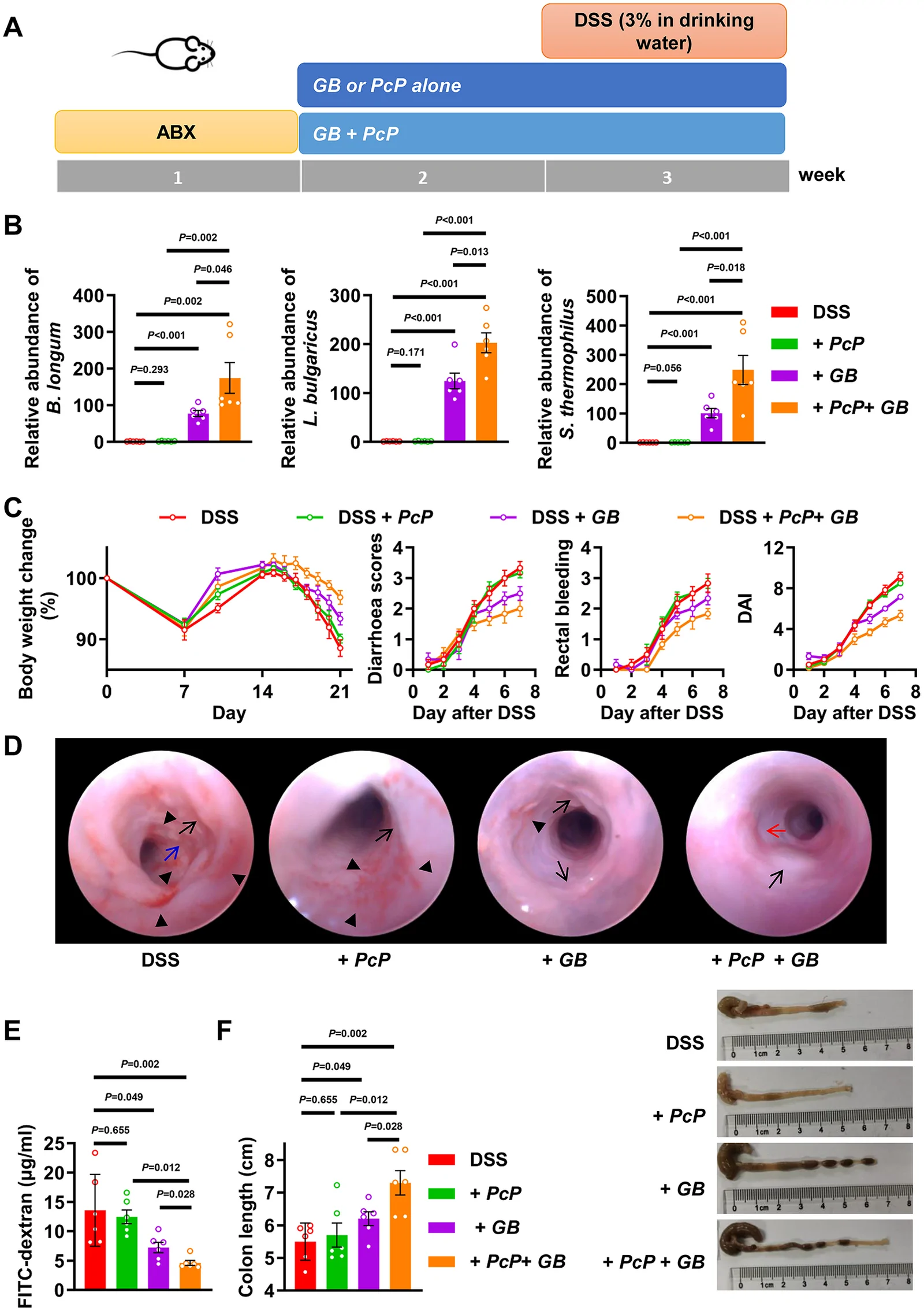
*Poria cocos* polysaccharide enhances the anti-colitic effect of probiotics on colitis in ABX mice. **(A)** Animal experimental design. Mice were given antibiotic cocktail for 1 week to deplete gut microbiota. Subsequently, mice were given 3% DSS in drinking water for 1 week to induce colitis, and were orally administered GB alone or in combination with PcP 1 week in advance. **(B)** Relative abundances of probiotics in mouse feces. **(C)** Body weight change, diarrhea and rectal bleeding scores, and DAI. **(D)**. Colonoscopic observation. Black triangles indicate hemorrhage; blue arrows indicate ulceration; black arrows indicate villous atrophy and fibrotic changes; red arrows indicate vasodilation. DAI and colonoscopy assess different aspects of colitis; near-normal endoscopic mucosa does not exclude mild residual inflammation detected by DAI. **(E)** Plasma FITC-dextran concentration. **(F)** Colon appearance and length. *N* = 6. PcP, *Poria cocos* polysaccharide. GB, Golden Bifid. ABX, antibiotic-treated.

**Figure 7:**
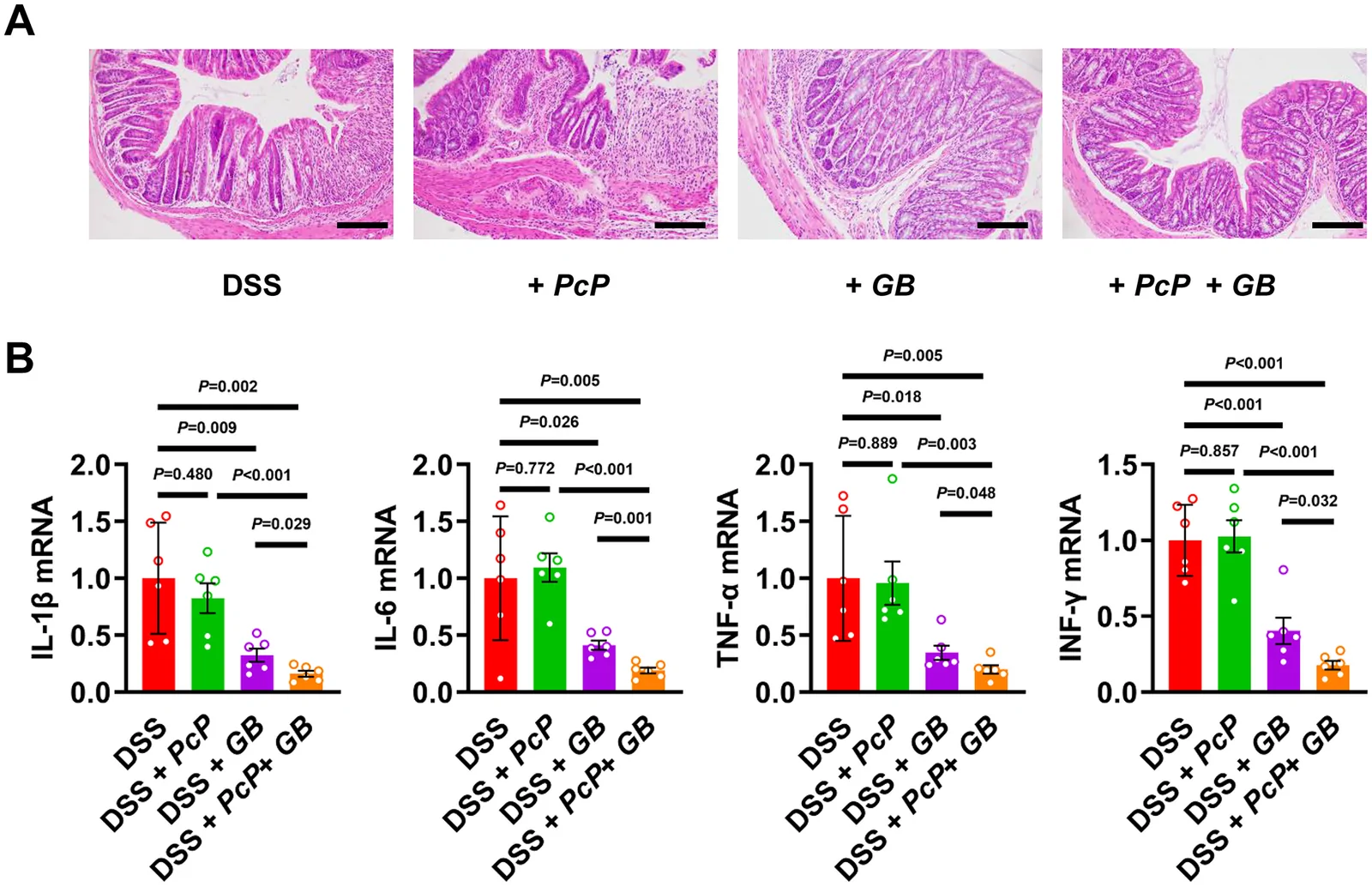
*Poria cocos* polysaccharide enhances the effect of probiotics in alleviating colonic pathological damage and inflammatory levels in ABX mice. **(A)** HE staining. Scale bars: 100 μm. Magnification: 400×. **(B)** mRNA levels of IL-1β, IL-6, TNF-α, and INF-γ in colon tissues. *N* = 6. PcP, *Poria cocos* polysaccharide; GB, Golden Bifid.

In contrast, the PcP alone treatment group failed to improve DSS-induced colitis, showing a similar degree of diarrhea, bleeding, and body weight loss to the DSS group, with no difference in DAI, and no improvement in colonic ulcers, hemorrhage, loose stools, as well as colonic villus structure, permeability, and inflammatory levels (Figures 6 and 7). This suggests that ABX abrogated the protective effect of PcP against colitis, and the efficacy of PcP is involved in gut microbiota.

Importantly, in ABX mice, the combined treatment of PcP and GB still achieved an enhanced anti-colitic effect. PcP promoted the efficacy of GB, resulting in milder diarrhea, bleeding, and body weight loss, a lower DAI, fewer colonic ulcers, hemorrhage, and loose stools, further recovery of colonic villi, decreased plasma FITC-dextran levels, further restoration of colon length, and lower colonic pathological damage and expression levels of inflammatory factors (IL-1β, TNF-α, IL-6, and INF-γ) compared with the GB alone treatment group (Figures 6 extbfand 7). These results clearly demonstrate that PcP promotes the anti-colitic effect of GB on colitis.

## 4 Discussion

In the present study, we systematically investigated the prebiotic potential of plant polysaccharides and their synergistic effects with GB in the amelioration of DSS-induced colitis in mice. Through systematic screening of 100 polysaccharides, we identified *Poria cocos* polysaccharide (PcP) as a potential prebiotic candidate that significantly promotes the proliferation, stress tolerance, and intestinal persistence of three core probiotic strains in GB, namely *Bifidobacterium longum, Lactobacillus bulgaricus*, and *Streptococcus thermophilus*. More importantly, we demonstrated that PcP enhances the anti-colitic efficacy of GB against colitis by strengthening its intestinal persistence rather than exerting a direct anti-inflammatory effect. These findings not only identify PcP as potential prebiotic candidate but also provide a feasible strategy to improve the clinical performance of probiotic preparations for inflammatory bowel disease (IBD).

PcP has long been recognized as a bioactive macromolecule with multiple pharmacological properties, including immunomodulatory, anti-inflammatory, and intestinal protective functions (Duan et al., 2023; Xu et al., 2023; Ye et al., 2022; Jiang et al., 2022; Song et al., 2025; Zhu et al., 2025). Previous studies have mainly focused on its direct regulatory effects on host immunity and intestinal epithelial cells (Duan et al., 2023; Wan et al., 2024; Gu et al., 2024). However, few studies have explored its potential as a prebiotic that specifically supports exogenous probiotics. In this study, by screening 100 natural polysaccharides, we found that PcP was the only candidate that simultaneously promoted the growth of all three probiotic strains in GB by more than 80%. Further *in vitro* experiments confirmed that PcP accelerated probiotic growth in a concentration-dependent manner and enhanced their resistance to simulated gastric acid and bile salts. To our knowledge, this study is the first to systematically characterize the effect of PcP on the proliferation and stress resistance of composite probiotic formulation GB. These results expand the current understanding of the biological functions of *Poria cocos* polysaccharide and support its application as a potential prebiotic candidate supplement.

A particularly meaningful finding is that PcP improves the anti-colitic effect of GB through a potential microbiota-associated mechanism. In pseudo-germ-free mice treated with broad-spectrum antibiotics, PcP alone failed to alleviate DSS-induced colitis, indicating that its protective effect against intestinal inflammation relies on the presence of gut microbiota (Wan et al., 2024). In contrast, GB still exerted anti-colitic effects in ABX mice, suggesting that GB retains its biological activity in antibiotic-treated mice with depleted indigenous microbiota. Notably, co-administration of PcP still enhanced the efficacy of GB in ABX mice, strongly indicating that PcP improves colitis mainly by boosting the persistence of the supplemented probiotics rather than by acting directly on the host. However, one limitation relates to the antibiotic cocktail (ABX) model. Despite achieving >99% depletion of total fecal bacteria, ABX may independently alter host intestinal physiology and DSS susceptibility, making it difficult to fully disentangle microbiota-dependent effects from direct antibiotic actions. In addition, our current findings only suggest microbiota involvement rather than proving microbiota dependence. Fecal microbiota transplantation (FMT) should be performed in future studies to provide more definitive evidence for the microbiota-dependent mechanism underlying PcP's protective effects.

Recent studies have shown that PcP mitigates colitis and drug-provoked intestinal injury by reshaping gut microbiota (Xu et al., 2023; Zhang et al., 2026; Zou et al., 2021; Tang et al., 2024), yet direct evidence for PcP acting on particular bacterial strains remains scarce. Our data demonstrate that PcP boosts *in-vitro* proliferation and *in-vivo* gut persistence of *B. longum, L. bulgaricus, and S. thermophilus*, though the exact active components and precise mechanisms remain incompletely resolved. As the three strains lack the full enzymatic toolkit to catabolize PcP-enriched high-molecular-weight β-1,3-D-glucan (Zhang et al., 2026), growth-promoting effects *in vitro* may stem from water-soluble heteropolysaccharide-derived oligosaccharide fragments, which can be processed by bacterial exo-acting glycoside hydrolases. This is consistent with the elevated proliferation and metabolic activity observed in our *in-vitro* assays (Figure 2). Besides serving as a fermentable carbon substrate, PcP may also exert non-nutritional physical effects, acting as an outer-surface material or adhesion matrix (Yang et al., 2022) to improve bacterial resistance to gastrointestinal stress and attachment to intestinal epithelium (Figure 2). Collectively, these *im-vitro* findings suggest several possible mechanisms linking PcP to enhanced bacterial adhesion, metabolic activity, gastrointestinal persistence, and anti-colitic outcomes. Nevertheless, these observations are derived from *in-vitro* assays and do not constitute demonstrated causal mechanisms for the effects observed *in vivo*.

Nevertheless, this study has several limitations that deserve further investigation. First, the PcP used in this study was a crude polysaccharide extract rather than a purified homogeneous component. The bioactive effects of crude PcP may arise from polysaccharides or co-extracted contaminants (e.g., proteins and lipids). Thus, the present findings are relevant to the crude PcP extract rather than a chemically defined, homogeneous polysaccharide. The precise structural basis underlying its potential prebiotic activity remains unclear and requires further purification, structural identification, and structure-activity relationship analysis. Second, we utilized strain-specific fecal qPCR to assess gastrointestinal persistence of administered probiotics. Nevertheless, fecal DNA-based qPCR cannot readily discriminate signals originating from stably engrafted viable bacteria vs. residual DNA derived from non-viable, transit-excreted cells. More robust evidence such as tissue-based FISH is warranted in future work to formally demonstrate mucosal attachment and stable engraftment. Third, although we demonstrated that PcP enhances probiotic persistence, we cannot fully exclude the possibility that PcP may indirectly modulate the host's immune system or intestinal microenvironment to support probiotic survival. Finally, the effects of PcP on the composition and function of the host's endogenous gut microbiota were not fully characterized. Future studies using 16S rRNA gene sequencing and metabolomic analysis will help clarify the global regulatory effects of PcP on the intestinal ecosystem. Another limitation of this study is that only male C57BL/6 mice were used for the *in vivo* experiments. Therefore, the present findings may not be directly generalizable to female mice.

## 5 Conclusion

The present study demonstrates that PcP functions as a potential prebiotic candidate that promotes the proliferation, stress resistance, and intestinal persistence of GB. PcP enhances the anti-colitic efficacy of GB against DSS-induced colitis by improving probiotic persistence in the gut. These findings provide a scientific basis for the development of novel prebiotic-probiotic combination preparations for the prevention and treatment of IBD.

## Data Availability

The raw data supporting the conclusions of this article will be made available by the authors, without undue reservation.
