# Supplementary material for: Poria cocos polysaccharide enhances the persistence and anti-colitic efficacy of three-strain probiotic preparation in DSS-induced colitis in mice
Source: Front Microbiol. 2026 Oct 29;17:1942804. doi: 10.3389/fmicb.2026.1942804 (PMC13757770; doi:10.3389/fmicb.2026.1942804)
Supplement: Supplementary file 1 [file Table_1.pdf]

Supplementary Tables and Figures

Supplementary Table 1. DSS consumption during model induction

Day	water	3% (w/v) DSS						
	-1	1	2	3	4	5	6	7
Figure 4								
Control	5.03	6.2	7.09	5.23	5.49	6.19	5.62	5.98
DSS	4.34	6.45	7.61	5.62	5.36	6.17	5.27	3.62
+ *PcP*	5.03	6.51	7.58	5.86	5.10	5.63	5.79	4.46
+ *GB*	4.17	6.39	8.15	5.12	5.3	5.62	5.21	4.10
+ *PcP* + *GB*	4.34	7.16	7.69	5.06	5.25	6.6	5.16	4.06
Figure 6								
DSS	5.48	6.51	7.37	4.66	5.6	6.00	5.51	3.38
+ *PcP*	4.88	6.57	6.38	5.44	5.98	6.13	6.35	3.72
+ *GB*	4.56	7.22	6.85	5.90	5.57	6.11	5.95	4.32
+ *PcP* + *GB*	5.18	6.51	7.09	5.44	5.05	6.12	5.39	4.86

Supplementary Table 2. Histopathological scoring criteria for HE-stained mouse colon sections

Parameter	Score	Description
Inflammation (0–3)	0	None
	1	Slight
	2	Moderate
	3	Severe
Crypt damage (0–5)	0	Normal
	1	Mild-to-moderate crypt loss (damage of basal 1/3 crypt)
	2	Severe crypt loss (damage of basal 2/3 crypt)
	3	Complete crypt loss with intact surface epithelium
	4	Partial loss of surface epithelium
	5	Entire crypt and surface epithelium loss
Ulceration (0–3)	0	Normal
	1	Mucosa destruction
	2	Muscularis mucosa destruction
	3	Submucosa destruction

Supplementary Table 3. qPCR primer sequences.

Species/Genes	Sequences		Product size (bp)	Amplification efficiency (%)
Bifidobacterium longum	Forward	TTCCAGTTGATCGCATGGTC	277	97.4
	Reverse	TCGCGCTTGCTCCCCGAT		
Lactobacillus bulgaricus	Forward	TACCAAGGCAATGATGCGTA	123	95.9
	Reverse	CATCAGACTTGCGTCCATTG		
Streptococcus thermophilus	Forward	GGAAATCCGTGACCTTC	137	98.2
	Reverse	CGTCAACAGTATTCATCAAG		
All Bacteria	Forward	ACTCCTACGGGAGGCAGCAG	198	98.9
	Reverse	ATTACCGCGGCTGCTGG		
Il-1β	Forward	TGCCACCTTTTGACAGTGATG	138	101.3
	Reverse	TGATGTGCTGCTGCGAGATT		
Il-6	Forward	GGGACTGATGCTGGTGACAA	90	97.4
	Reverse	ACAGGTCTGTTGGGAGTGGT		
Tnf-α	Forward	TAGCCCACGTCGTAGCAAAC	99	99.9
	Reverse	TGTCTTTGAGATCCATGCCGT		
Gapdh	Forward	CTTCTCCTGCAGCCTCGT	139	102.6
	Reverse	TCATCCACCTCCCCACAGTA		

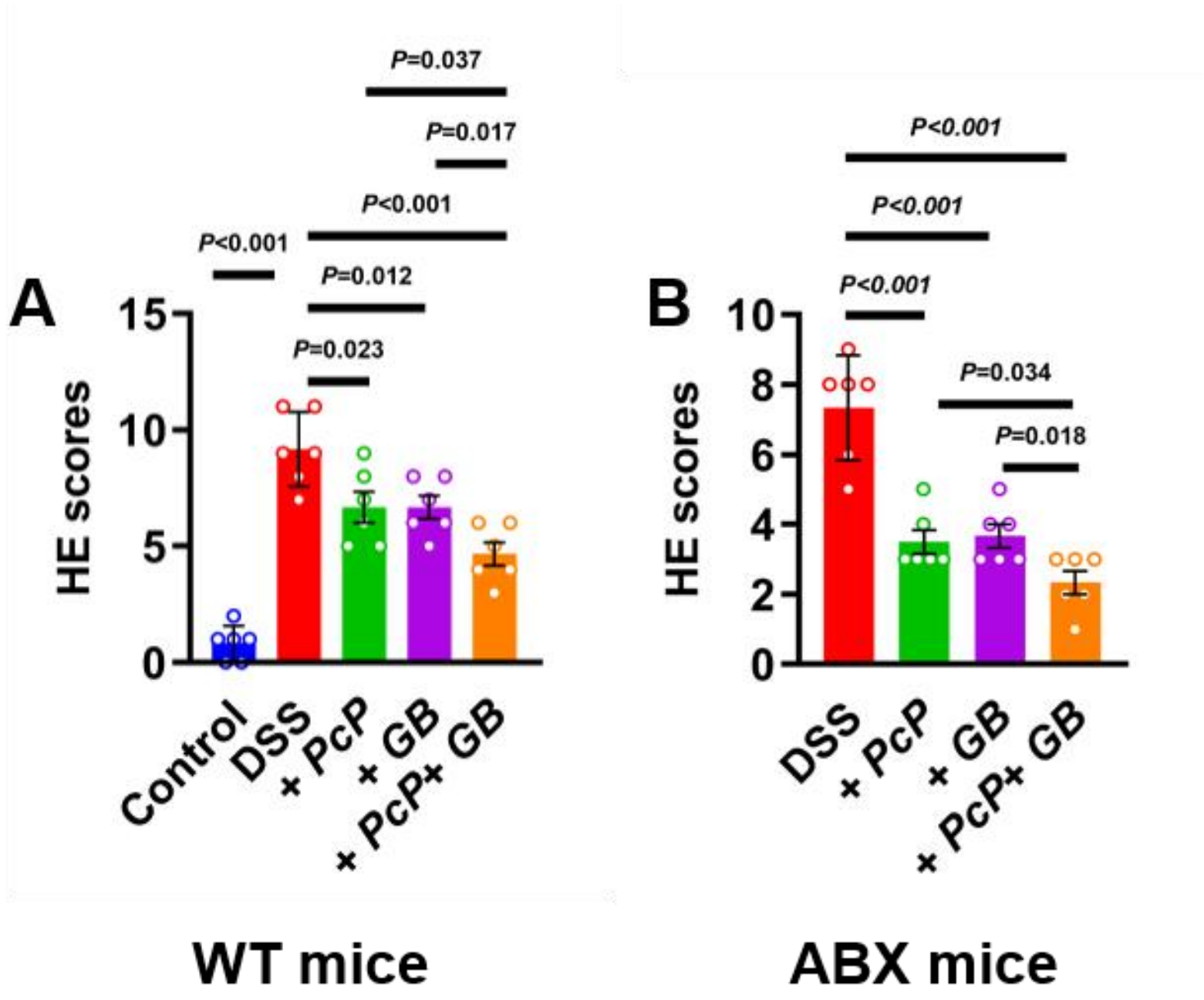

Supplementary Figure 1. Quantitative histopathological scores related to Figure 5A (WT mice) and Figure 7A (ABX mice)

N=6. PcP, Poria cocos polysaccharide; GB, Golden Bifid.
